# Characterization of Outer Membrane Proteome of *Akkermansia muciniphila* Reveals Sets of Novel Proteins Exposed to the Human Intestine

**DOI:** 10.3389/fmicb.2016.01157

**Published:** 2016-07-26

**Authors:** Noora Ottman, Laura Huuskonen, Justus Reunanen, Sjef Boeren, Judith Klievink, Hauke Smidt, Clara Belzer, Willem M. de Vos

**Affiliations:** ^1^Laboratory of Microbiology, Wageningen UniversityWageningen, Netherlands; ^2^Metapopulation Research Centre, University of HelsinkiHelsinki, Finland; ^3^Department of Veterinary Biosciences, University of HelsinkiHelsinki, Finland; ^4^Microbiology and Biotechnology, Department of Food and Environmental Sciences, University of HelsinkiHelsinki, Finland; ^5^Laboratory of Biochemistry, Wageningen UniversityWageningen, Netherlands; ^6^Immunobiology, Department of Bacteriology and Immunology, and Research Programs Unit, University of HelsinkiHelsinki, Finland

**Keywords:** *Akkermansia muciniphila*, outer membrane, gut microbiota, proteomics, pili, PVC superphylum

## Abstract

*Akkermansia muciniphila* is a common member of the human gut microbiota and belongs to the Planctomycetes-Verrucomicrobia-Chlamydiae superphylum. Decreased levels of *A. muciniphila* have been associated with many diseases, and thus it is considered to be a beneficial resident of the intestinal mucus layer. Surface-exposed molecules produced by this organism likely play important roles in colonization and communication with other microbes and the host, but the protein composition of the outer membrane (OM) has not been characterized thus far. Herein we set out to identify and characterize *A. muciniphila* proteins using an integrated approach of proteomics and computational analysis. Sarkosyl extraction and sucrose density-gradient centrifugation methods were used to enrich and fractionate the OM proteome of *A. muciniphila*. Proteins from these fractions were identified by LC-MS/MS and candidates for OM proteins derived from the experimental approach were subjected to computational screening to verify their location in the cell. In total we identified 79 putative OM and membrane-associated extracellular proteins, and 23 of those were found to differ in abundance between cells of *A. muciniphila* grown on the natural substrate, mucin, and those grown on the non-mucus sugar, glucose. The identified OM proteins included highly abundant proteins involved in secretion and transport, as well as proteins predicted to take part in formation of the pili-like structures observed in *A. muciniphila*. The most abundant OM protein was a 95-kD protein, termed PilQ, annotated as a type IV pili secretin and predicted to be involved in the production of pili in *A. muciniphila*. To verify its location we purified the His-Tag labeled N-terminal domain of PilQ and generated rabbit polyclonal antibodies. Immunoelectron microscopy of thin sections immunolabeled with these antibodies demonstrated the OM localization of PilQ, testifying for its predicted function as a type IV pili secretin in *A. muciniphila*. As pili structures are known to be involved in the modulation of host immune responses, this provides support for the involvement of OM proteins in the host interaction of *A. muciniphila*. In conclusion, the characterization of *A. muciniphila* OM proteome provides valuable information that can be used for further functional and immunological studies.

## Introduction

*Akkermansia muciniphila* is a Gram-negative, anaerobic bacterium, which colonizes the mucus layer of the human gastrointestinal (GI) tract (Derrien et al., 2004). *A. muciniphila* is considered to be an important member of the GI microbiota, because of the inverse correlation between its abundance and several intestinal disorders, including inflammatory bowel diseases and obesity (Png et al., 2010; Karlsson et al., 2012; Rajilic-Stojanovic et al., 2013). Moreover, experiments with germ-free mice mono-associated with *A. muciniphila*, or conventional mice on a high fat diet that are fed *A. muciniphila*, have shown that *A. muciniphila* plays a role in host immune response, restoration of mucus layer thickness and mucus production (Derrien et al., 2011; Everard et al., 2013; Shin et al., 2014). In addition, extracellular vesicles from *A. muciniphila* were shown to have protective effects on the development of dextran sulfate sodium (DSS) induced colitis in mice (Kang et al., 2013). Finally, *A. muciniphila* has also been shown to adhere to intestinal epithelium and improve enterocyte monolayer integrity of Caco-2 cells (Reunanen et al., 2015). These findings suggest important host-bacteria interactions, the mechanisms of which are yet to be discovered (see Derrien et al., 2016 for a recent review).

Bacterial outer membrane (OM) proteins play important roles in communication with other microbes and the host, as well as in colonization and substrate transport (Tseng et al., 2009; Galdiero et al., 2012). Subcellular fractionation techniques combined with mass spectrometry-based proteomic analysis are powerful tools for identifying proteins in different bacterial compartments. These techniques have been successfully used for studying the protein composition of intestinal bacteria, such as the OM of *Bacteroides fragilis* and *Bacteroides thetaiotaomicron* (Elhenawy et al., 2014; Wilson et al., 2015), surface proteins of *Propionibacterium freudenreichii* (Le Marechal et al., 2015) and OM vesicles of *Escherichia coli* Nissle 1917 (Aguilera et al., 2014).

*Akkermansia muciniphila* is a member of the Planctomycetes-Verrucomicrobia-Chlamydiae (PVC) superphylum, which contains bacteria from several groups and various environments with different lifestyles (Wagner and Horn, 2006; Gupta et al., 2012; Kamneva et al., 2012). Bacteria from this superphylum were previously suggested to have a compartmentalized cell plan with a cytoplasmic membrane as the outermost membrane, and an intracytoplasmic membrane containing a condensed nucleoid and ribosomes (Lee et al., 2009). However, these observations have been challenged by more recent data suggesting that the PVC cell plan is actually a variation, not an exception, of the Gram-negative cell plan, and that the bacteria have an outer and an inner membrane (IM) with possible invaginations of the IM inside the cytoplasm (Devos, 2014).

There is limited information available on the membrane structure and composition of *A. muciniphila*, and most reports have focused on *in silico* analysis of Verrucomicrobia membranes, instead of experimental approaches (Santarella-Mellwig et al., 2010; Kamneva et al., 2012; Speth et al., 2012). Recently, the proteome of a termite hindgut representative of the Verrucomicrobia, *Diplosphaera colotermitum* TAV2, was experimentally studied, but this report did not focus on membrane proteins (Isanapong et al., 2013). The presence of OM biomarkers, including genes involved in lipopolysaccharide (LPS) insertion, in the genome of *A. muciniphila* was confirmed computationally (Speth et al., 2012). We have experimentally verified the presence of LPS in *A. muciniphila* (Ottman, 2015). No genes coding for membrane coat-like proteins were found in *A. muciniphila*, unlike in some other Verrucomicrobia (Santarella-Mellwig et al., 2010).

In the present study, we set out to identify and characterize *A. muciniphila* proteins using an integrated approach of proteomics and computational analysis. Successful extraction of OM proteins was established, and the proteins were identified with liquid chromatography-tandem mass spectrometry (LC-MS/MS). The abundance of *A. muciniphila* proteins in the OM fraction was compared to the whole proteome of *A. muciniphila* and a fraction enriched for intracellular proteins. Candidates for OM proteins derived from the proteomics analysis were subjected to computational screening to verify their location in the cell. The OM location of the most abundant membrane protein, termed PilQ, in *A. muciniphila* was confirmed by immunoelectron microscopy. PilQ is predicted to be a secretin involved in the production of type IV pilins, long surface exposed filaments involved in a variety of functions, including motility and adherence to host cells, and found in virtually all prokaryotes, including the PVC superphylum (Berry and Pelicic, 2015). We also explored the presence of these proteins as a function of the growth on mucus, resulting in the identification of several OM proteins related to mucosal colonization. The results indicate that *A. muciniphila* produces OM proteins involved in secretion and transport in high abundance, and these, including PilQ, may be involved in its capacity to interact with the host.

## Materials and Methods

### Bacterial Growth Conditions

*Akkermansia muciniphila* Muc^T^ (ATTC BAA-835) was grown in a basal medium as described previously, except without the addition of rumen fluid (Derrien et al., 2004). The medium was supplemented with either hog gastric mucin (0.5 %, Type III; Sigma-Aldrich, St. Louis, MO, USA) purified by ethanol precipitation as described previously (Miller and Hoskins, 1981), or D-glucose (10 mM, Sigma–Aldrich). The medium with glucose was also supplemented with Bacto^TM^ casitone (BD, Sparks, MD, USA), BBL^TM^ yeast extract (BD), tryptone (Oxoid Ltd, Basingstoke, Hampshire, England), peptone (Oxoid ltd) (2 g/l each) and L-threonine (Sigma–Aldrich) (2 mM). Incubations were performed in serum bottles sealed with butyl-rubber stoppers at 37°C under anaerobic conditions provided by a gas phase of 182 kPa (1.5 atm) N_2_/CO_2_. Growth was measured by following optical density at 600 nm (OD600) using a spectrophotometer.

### Bacterial Fractionation Methods

Membrane proteins of *A. muciniphila* were isolated from liquid cultures with two different methods, using either *N*-lauroyl- sarcosine (sarkosyl) or sucrose density-gradient centrifugation, as described previously, with minor modifications (Hobb et al., 2009).

Briefly, for sarkosyl treatments, 250 ml cultures of *A. muciniphila* were grown on mucin for 16 h (OD_600_ = 1.56) or on glucose for 40 h (OD_600_ = 0.34). Cells were harvested, washed twice with phosphate buffered saline (PBS) and resuspended in 9 ml 10 mM HEPES, pH 7.4, and lysed by passing the culture three times through a French press (Aminco, American Instrument Co., Inc., Silver Spring, Maryland, USA) at 1000 psi (40 K cell). The lysed cell preparation was centrifuged at 10 000 *g* for 10 min at 4°C to remove cell debris and unlysed cells. The membranes were collected by ultracentrifugation of the supernatant at 100 000 *g* for 1 h at 4°C. The supernatant was collected and stored at -20°C. This sample was later analyzed by LC-MS/MS as the intracellular fraction. The pellet was resuspended in 2 ml 10 mM HEPES, pH 7.4, washed in a total volume of 10 ml 10 mM HEPES, pH 7.4, and spun again in the ultracentrifuge (using the conditions described above). The pellet was resuspended in 5 ml 1 % (w/v) *N*-lauroylsarcosine (sarkosyl) (Sigma–Aldrich) in 10 mM HEPES, pH 7.4, and incubated at 37°C for 30 min with shaking to solubilize cytoplasmic membranes. The sarkosyl-treated membranes were spun at 100 000 *g* for 1 h at 4°C and the pellet was washed with 7 ml 10 mM HEPES, pH 7.4. Following the final ultracentrifugation, the pellet containing the OM fraction was resuspended in 1 ml 10 mM HEPES, pH 7.4 and stored at -20°C.

For the sucrose-density gradient centrifugation treatments, 250 ml cultures of *A. muciniphila* were grown on mucin for 16 h (OD600 = 1.51) or on glucose for 40 h (OD600 = 0.35). Cells were harvested, washed twice with phosphate buffered saline (PBS) and resuspended in 7 ml 10 mM HEPES, pH 7.4, and lysed by passing the culture three times through a French press at 1000 psi (40K cell). The lysed cells preparation was centrifuged at 10 000 *g* for 10 min at 4°C. The supernatant was ultracentrifuged at 100 000 *g* for 60 min at 4°C to pellet the total membranes. The membrane pellet was washed in 10 ml 10 mM HEPES, 0.05 M EDTA pH 7.5 (HE buffer) and ultracentrifuged again. The final membranes were homogenized in 2 ml HE buffer. Continuous sucrose gradients were prepared by layering sucrose solutions (prepared in HE buffer) into 14 × 95 mm polyallomer ultracentrifuge tubes (Seton Scientific, Petaluma, CA, USA) in the following order: 0.4 ml 60% (w/v), 0.9 ml 55%, 2.2 ml 50%, 2.2 ml 45%, 2.2 ml 40%, 1.3 ml 35% and 0.4 ml 30%. Total membranes were layered on top of each gradient, with no more than 2.5 ml per gradient. Sucrose gradients were centrifuged in a TST 41.14-41000 RPM swinging-bucket rotor (Kontron) at 250 000 g for 16 h at 4°C. The sucrose gradient tubes were then removed from the rotor buckets and 500 μl fractions (24 fractions for each gradient) were collected from the bottom of each gradient by puncturing the tube with a needle and allowing the sample to drip out by gravity. The samples were stored in 2 ml low binding tubes (Eppendorf, Hamburg, Germany) at -20°C.

For comparison, the whole proteome fraction was obtained from *A. muciniphila* cultures grown with mucin or glucose as the carbon source. Bacterial cells from an overnight 2 ml culture were spun down, washed with PBS and suspended into 500 μl of PBS. Cells were lysed by sonication, using a Branson sonifier equipped with a 3 mm tip (four pulses of 20 s with 30 s rest on ice in-between each pulse, strength of the pulse was 4). The samples were stored in 2 ml low binding tubes (Eppendorf, Hamburg, Germany) at -20°C.

### Protein Identification by Mass Spectrometry

To determine the protein content of cell extracts, the Qubit^®^ Protein Assay Kit (Life technologies, Eugene, OR, USA) was used according to the manufacturer’s instructions. Samples were loaded on a 10% acrylamide separation gel (25201, Precise^TM^ Protein Gels, Thermo Scientific, Rockford, IL, USA) using the mini-PROTEAN 3 cell (Bio-Rad Laboratories, Hercules, CA, USA). The electrophoresis procedure was according to the manufacturer’s instructions. Gels were stained using Coomassie Brilliant Blue (CBB) R-250 as indicated in the protocol of the mini-PROTEAN 3 system.

In-gel digestion of proteins and purification of peptides were done following a modified version of a previously described protocol (Rupakula et al., 2013). Disulphide bridges in proteins were reduced by covering whole gels with reducing solution (10 mM dithiothreitol, pH 7.6, in 50 mM NH_4_HCO_3_), and the gels were incubated at 60°C for 1 h. Alkylation was performed for 1 h by adding 25 ml of iodoacetamide solution (10 mM iodoacetamide in 100 mM Tris-HCl, pH 8.0). Gels were thoroughly rinsed with demineralized water in between steps. Each of the used gel lanes was cut into either five slices (sarkosyl-extracted OM, intracellular proteins and whole proteome) or one slice (sucrose density-gradient centrifugation samples), and the slices were cut into approximately 1 mm × 1 mm × 1 mm cubes and transferred to separate 0.5 ml protein LoBind tubes (Eppendorf, Hamburg, Germany). Enzymatic digestion was done by adding 50 μl of trypsin solution (5 ng/μl trypsin in 50 mM NH_4_HCO_3_) to each tube, and by incubating at room temperature overnight with gentle shaking. Extraction of peptides was performed with manual sonication in an ultrasonic water bath for 1 s before the supernatant was transferred to a clean protein LoBind tube. Trifluoroacetic acid (10%) was added to the supernatant to reach a pH between 2 and 4. The supernatant was used for LC-MS/MS analysis. Samples were measured by nLC–MS/MS with a Proxeon EASY nLC and a LTQ-Orbitrap XL mass spectrometer as previously described (Lu et al., 2011).

LC–MS data analysis was performed as described previously (Rupakula et al., 2013) with false discovery rates (FDRs) set to 0.01 on peptide and protein level, and additional result filtering (minimally 2 peptides necessary for protein identification of which at least one is unique and at least one is unmodified). To analyze the abundance of proteins in the fractions, their label-free quantification (LFQ) intensities was compared (Cox et al., 2014). Non-existing LFQ intensity values due to not enough quantified peptides were substituted with a value lower than the LFQ intensity value for the least abundant, quantified protein. The mass spectrometry proteomics data have been deposited to the ProteomeXchange Consortium via the PRIDE (Vizcaino et al., 2016) partner repository with the dataset identifier PXD004400.

### Enzyme Activity Assay

Enzyme activity was tested in the OM and intracellular fractions by incubating them at 37°C with colorimetric or fluorimetric substrates, as described previously (Rosendale et al., 2012). The following substrate/enzyme combinations were used: 4-nitrophenyl *N*-acetyl-β-D-glucosaminide/*N*-acetyl-β-glucosaminidase, *p*-nitrophenyl-alpha-L-fucoside/fucosidase, PNP *N*-acetyl-glucosamine-sulfate/GlcNAc-sulfatase, and 2′-(4-methylumbelliferyl)-α-D-*N*-acetylneuraminic acid/sialidase. Substrates were purchased from Carbosynth (Compton, Berkshire, UK). Reaction volume was 20 μl and substrate concentration was 1 mM in each reaction. All reactions were performed in duplicate.

### Bioinformatics Analysis

Proteins were categorized based on results of SignalP v.4.0 (set for Gram-negative bacteria) (Petersen et al., 2011), LipoP v.1.0 (Juncker et al., 2003), SecretomeP v.2.0 (set for Gram-negative bacteria) (Bendtsen et al., 2005), TMHMM v.2.0 (Krogh et al., 2001) and BOMP (Berven et al., 2004). Subcellular localization was determined using CELLO v.2.5 (set for Gram-negative bacteria) (Yu et al., 2004) and PSORTb v3.0 (set for Gram-negative bacteria) (Yu et al., 2010). BLAST searches were run against the non-redundant (nr) database at http://blast.ncbi.nlm.nih.gov/ using default settings. TIGRFAM and Pfam were used for screening proteins with PEP-CTERM domains (Selengut et al., 2007; Finn et al., 2014).

### Overproduction and Purification of Amuc_1098A Protein

The DNA coding for the N-terminal half of PilQ, the product of the gene with locus tag Amuc_1098, was amplified by PCR from the chromosomal DNA of *A. muciniphila* using a primer pair containing NdeI site (5′ATA*CATATG*GATGGCGGCGCCGTCGGAACCTC; restriction site in italics) and XhoI site (5′ATA*CTCGAG*GGGCTTAAGGCCGGAGGAGCTTT; restriction site in italics) (Sigma–Aldrich). The obtained PCR product was digested with NdeI and XhoI and cloned into similarly digested pET26b vector (Novagen) and transformed into *E. coli* TOP10 (Invitrogen). Agarose gel electrophoresis and sequencing were used to verify the right insert and the obtained plasmid (termed pAmuc_1098A) was subsequently transformed into the expression strain *E. coli* BL21 (DE3) (Invitrogen) containing plasmid pSJS1240 (Kim et al., 1998) and transformants were selected for resistance to kanamycin and spectinomycin (both at 50 μg/ml). Protein production was induced in an exponentially growing aerobic culture at optical density at 600 nm of approximately 0.5 by adding isopropyl β-d-1-thiogalactopyranoside (IPTG) to a final concentration of 1 mM. After a further overnight incubation at 37°C, cells were collected and lysed by sonication and the protein, referred to as P-Amuc_1098A, was purified under native conditions using Ni-NTA His Bind Resin (Novagen) according to manufacturer’s instructions. The purity was tested with sodium dodecyl sulfate-polyacrylamide gel electrophoresis (SDS-PAGE).

### Generation of Polyclonal Antibody against P-Amuc_1098A Protein

Polyclonal rabbit antibodies were produced against P-Amuc_1098A protein with an immunization protocol as described previously (Johnston et al., 1991), at the Laboratory Animal Centre of the University of Helsinki. In short, a rabbit was immunized four times with 3 weeks interval for each injection. Pre-immunization sera were collected before initiating the immunization. In the first immunization, purified P-Amuc_1098A protein (192 μg) was mixed with an equal amount of Freund’s complete adjuvant. For the subsequent immunizations (booster injections), P-Amuc_1098A protein (96 μg) was diluted 1:1 volume in Freund’s incomplete adjuvant and injected into rabbit. Blood was collected from the rabbit 10 days after the last booster injection. The blood was allowed to clot for 1-2 h at 37°C after which the blood clot was separated from the serum by centrifugation at 3 000 *g* for 10 min. Then the serum was divided into aliquots and stored at -80°C prior to use.

### Immunogold Labeling and Electron Microscopy of Thin Sections

Immunogold labeling of *A. muciniphila* thin sections was performed as described previously with minor changes (Reunanen et al., 2012). In short, *A. muciniphila* cells were grown in mucin medium for two overnights and washed once with phosphate buffer (0.1 M Na-phosphate, pH 7.4) before fixation. The fixed cells were embedded in Lowicryl HM20 resin, 50 nm thin sections were cut from polymerized Lowicryl and mounted on nickel grids. Grids containing the *A. muciniphila* thin sections were treated with anti-P-Amuc_1098A rabbit immune serum (1:5 dilution), and subsequently with protein A conjugated to 10 nm diameter gold particles. Finally, the grids were post-stained with uranyl acetate and lead citrate using an UltroStain apparatus (Leica). The grids were examined and images obtained with a JEOL JEM-1400 transmission electron microscope (Jeol Ltd., Tokyo, Japan).

### Statistical Analysis

Statistical analysis of the results from the enzyme activity assay was performed by one-way analysis of variance (ANOVA) using IBM SPSS software (IBM SPSS Statistics 22); *p* values <0.05 were considered significant.

## Results

**FIGURE 1:**
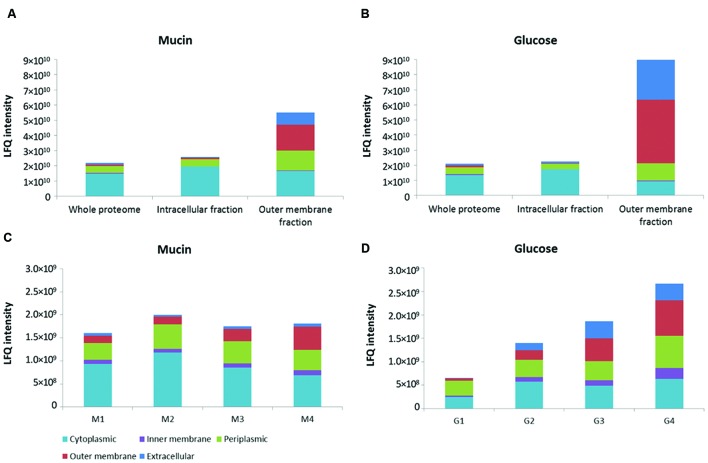
**Distribution of proteins from different bacterial compartments in isolated fractions.** OM fraction isolated from sarkosyl-extracted membranes in comparison to whole proteome and intracellular fraction of both mucin and glucose-grown cells of *A. muciniphila* **(A,B)**. Fractions of sucrose density-gradient centrifugation (see Supplementary Figure S1) of membranes of *A. muciniphila* grown on mucin or glucose **(C,D)**. Label-free quantification (LFQ) intensity was used as a proxy for absolute protein abundance. CELLO software was used to predict protein localization.

*Akkermansia muciniphila* OM proteins were isolated using two different methods, based on either selective sarkosyl extraction or sedimentation gradient centrifugation of membrane proteins, derived from cells grown with mucin or glucose as major carbon and energy sources (**Figure 1**). Label-free mass spectrometry of the trypsin-treated fractions was performed to identify the extracted proteins in comparison with those recovered from total cells (whole proteome). In addition, the location of the identified proteins was predicted by using the CELLO software. To determine the abundance of proteins within the fractions from different cellular locations, we compared their LFQ intensities as these are commonly used as a proxy for absolute protein abundance in mass spectrometry data analysis (Cox et al., 2014).

An over 20-fold enrichment of predicted OM proteins was achieved in the sarkosyl-treated membranes in comparison with the whole proteome and intracellular fraction of both mucin and glucose-grown cells of *A. muciniphila* (**Figures 1A,B**). Moreover, the predicted periplasmic and extracellular proteins were slightly enriched in the OM fraction at the expense of cytoplasmic proteins that were enriched in the intracellular fraction.

Complementary to the sarkosyl treatment procedure, we applied sucrose density gradient centrifugation of *A. muciniphila* membrane fractions obtained from cells grown on mucin or glucose. Analysis of the recovered proteins from each fraction revealed different protein abundance in the mucin versus glucose-grown cell membranes, likely reflecting the adaptation to the different environmental conditions (Supplementary Figure S1). The abundance of OM proteins was the lowest in the fraction with the lowest sucrose density, and increased along the gradient for proteins derived from both growth conditions (**Figures 1C,D**). However, the enrichment of predicted OM proteins was not more than a factor of 2–5 by the sucrose gradient centrifugation and hence it was evident that the sarkosyl treatment procedure was superior (**Figures 1C,D**).

**FIGURE 2:**
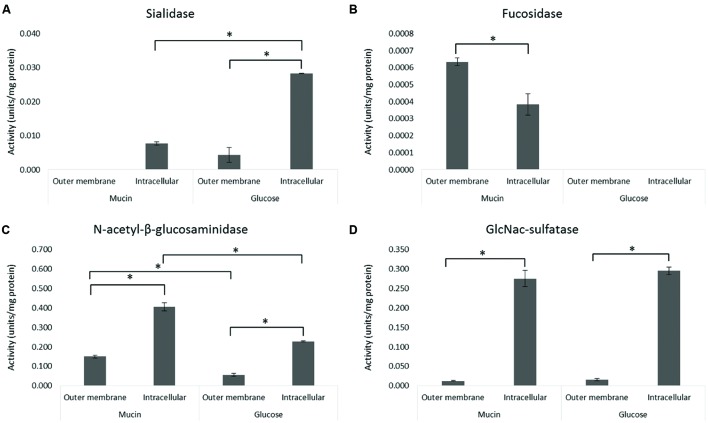
**Activity of mucin-degrading enzymes in the OM and intracellular fractions.** The enzyme activity of sialidase **(A)**, fucosidase **(B)**, *N*-acetyl-β-glucosaminidase **(C)**, and GlcNAc-sulfatase **(D)** was determined from OM and intracellular (including periplasmic space) fractions of *A. muciniphila* grown on mucin or glucose as described in **Figure 1**. Average of duplicate measurements is shown. Significant differences (*p* < 0.05) in the enzyme activities between samples are indicated with an asterisks.

The sarkosyl-treated OM fraction was compared with the intracellular fraction derived from the same *A. muciniphila* cells (**Figures 1A,B**) for the activity of four relevant mucin-degrading enzymes (sialidase, fucosidase, *N*-acetyl-β-glucosaminidase, GlcNAc-sulfatase) (**Figure 2**). Except for the fucosidase activity, it appeared that the sialidase, *N*-acetyl-β-glucosaminidase and GlcNAc-sulfatase activity was significantly higher in the intracellular than the OM fractions, for both glucose and mucin-grown cells (**Figures 2A,C,D**). As none of the candidate enzymes for these activities was predicted to be located in the OM, this supported the purification of OM proteins by the sarkosyl treatment. In contrast, most candidate proteins were predicted to carry a signal sequence and hence their presence in the intracellular fraction indicated that they are at least partly among the periplasmic space proteins that are also found in this fraction (**Figures 1A,B**). Different results were obtained for the fucosidase activity (**Figure 2B**). This enzymatic activity was enriched in the OM fraction, which is compatible with the experimentally verified OM location of one of the fucosidases (encoded by the gene with locus tag Amuc_0146; see below). Remarkably, this fucosidase activity was strongly induced in the extracts of *A. muciniphila* grown on mucin, which is in line with the production of 1,2-propanediol from fucose during growth on mucin but not on glucose (Ottman, 2015).

### Analysis of the *A. muciniphila* Proteome

**FIGURE 3:**
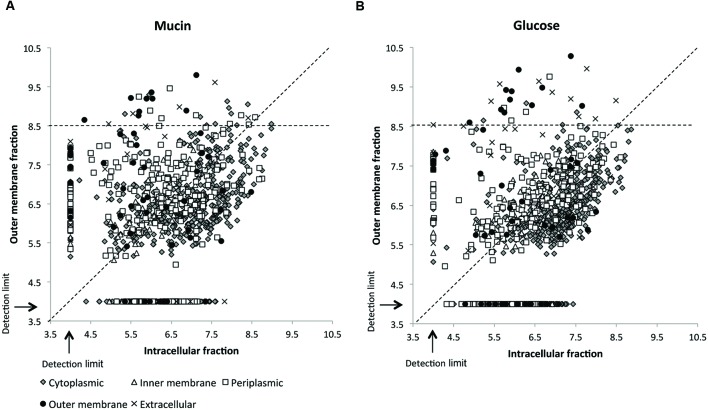
**Sarkosyl extraction leads to enrichment of OM and intracellular proteins.** Subcellular localization of proteins identified from the OM and intracellular fraction of *A. muciniphila* grown on mucin **(A)** or glucose **(B)**. CELLO was used to predict protein localization. Relative abundances of the proteins are presented on a log10 scale with the dashed diagonal line indicating an even distribution between OM and intracellular fraction. A relative abundance of 4.0 represents proteins that were not detected or were under the detection limit. All proteins with LFQ intensity above 8.5 in the sarkosyl-extracted OM fraction (horizontal dashed line) were included in the preliminary list of potential OM proteins.

While over 1000 different proteins could be identified in the total *A. muciniphila* proteome, the number of sarkosyl-treated OM proteins amounted to slightly lower amounts (812 and 719 proteins from the mucin and glucose grown cells, respectively) but exceeded those found in the sucrose gradient fractions (up to ~500 proteins), indicative of the efficiency of the detergent treatment (Supplementary Table S1). Of further interest was the comparison of the protein abundancies of these sarkosyl-treated OM proteins and the intracellular proteins obtained from the same *A. muciniphila* cells, grown on either mucin or glucose (**Figure 3**). The resulting comparative proteome plots showed that many of the predicted OM proteins were highly abundant in the OM fraction with a relative LFQ intensity above 8.5. Remarkably, several predicted extracellular, periplasmic and cytoplasmic proteins (12, 12, and 14, respectively) were also similarly abundant and enriched in the OM fraction in *A. muciniphila* cells grown on either mucin or glucose. Hence, all the proteins with an LFQ intensity above 8.5 and proteins predicted as OM proteins by CELLO or PSORTb (a total of 145) were further analyzed as described below.

### Identification, Annotation and Classification of *A. muciniphila* OM Proteins

Next, we confirmed the predicted localization of the 145 proteins that were experimentally found to be enriched in the OM fraction by applying several criteria, supported by advanced bioinformatic predictions: a protein was considered as a potential OM protein if (1) both CELLO and PSORTb programs predicted the protein to be localized in the OM, (2) the LipoP software predicted the protein to be a lipoprotein, (3) BOMP software predicted the presence of beta-barrel(s), or (4) the protein had no signal protein, but SecretomeP predicted unclassical secretion (Supplementary Figure S2). Proteins that had a signal peptide but did not fulfill any of the above criteria were assumed to be periplasmic. After applying these criteria, 73 proteins from mucin-grown cells and 76 proteins from glucose-grown cells in the isolated OM fractions were predicted to be true OM proteins. Moreover, 5 out of the 14 cytoplasmic and 9 out of the 12 periplasmic proteins included in the preliminary list were identified as true OM proteins. In total, 79 truly predicted OM and membrane-associated extracellular proteins were identified (Supplementary Table S2). It is of interest to note that the majority of these were also present in the sucrose density-gradient samples: 62 proteins for the mucin-grown and 58 proteins for the glucose-grown condition (data not shown).

Additionally, we searched for potential OM proteins, which were not present in either of the sarkosyl-extracted protein fractions. The *A. muciniphila* genome was found to encode additional 17 proteins, which were predicted as OM proteins according to the applied criteria (see above), and 12 (71%) of these were uncharacterized proteins (Supplementary Table S3). Two of the proteins (products of genes tagged as Amuc_0356 and Amuc_0480) were found to be present in the sucrose density-gradient fractions. Three of the proteins (products of genes tagged as Amuc_0983, Amuc_1011 and Amuc_1945) were present in low amounts when the whole proteome of *A. muciniphila* was analyzed. However, all these proteins are likely to be produced, since RNA-seq analysis of *A. muciniphila* grown on mucin or glucose confirmed the production of transcripts for the corresponding 17 genes (Ottman, 2015).

The proteins identified as true OM proteins had multiple and different functions (Supplementary Figure S3). Nine proteins obtained from cells grown at both conditions were OM proteins containing an autotransporter barrel domain. OM proteins enriched during growth on mucin (total of four) or on glucose (total of three) were found to be involved in RND (Resistance-Nodulation-Division) eﬄux systems. Mucin-grown cells contained three and glucose-grown cells two YD repeat proteins. The function of many of the predicted OM proteins was unknown (26 proteins for mucin-grown and 29 for glucose-grown cells). BLAST searches were performed for all uncharacterized proteins with LFQ intensity above 8.5, but this did not reveal any closely related proteins.

### Influence of Environmental Growth Conditions on OM Protein Abundancies

From the identified OM proteins, 23 out of the 79 showed more than a 10-fold change in abundancy between *A. muciniphila* grown on either mucin or glucose (Supplementary Table S2). Six out of the nine proteins containing an autotransporter barrel domain were present at a higher level in glucose-grown as compared to mucin-grown cells. In addition, a two-component regulator propeller domain protein and a peptidase were more abundant in mucin-grown cells, whilst a glycoside hydrolase family 16 protein and peptidyl-prolyl *cis-trans* isomerase were present in higher abundance in glucose-grown cells (products of genes with locus tags Amuc_0371 and Amuc_0576 as well as Amuc_2108 and Amuc_1026, respectively). Many uncharacterized proteins were among the ones with highest fold-change differences between the two conditions. There were four uncharacterized proteins (products of genes with locus tags Amuc_0789, Amuc_0837, Amuc_1333, Amuc_2165) exclusively found in the OM fraction extracted from glucose-grown cells and one uncharacterized protein (encoded by the gene with locus tag Amuc_0006), exclusively found in mucin-grown cells.

BLAST searches and subsequent TIGRFAM and PFAM analyses revealed that three of the glucose-exclusive proteins in the OM fraction contained a PEP-CTERM domain (Supplementary Table S4). This domain is thought to be involved in protein sorting and cell surface localization (Haft et al., 2006). The domain includes the motif Pro-Glu-Pro (PEP), which is considered a potential recognition or processing site, followed by a predicted transmembrane helix and a cluster rich in basic amino acids. These target proteins are generally destined to transit cellular membranes during their biosynthesis and undergo further posttranslational modifications, such as glycosylation. PEP-CTERM domains have been first identified in the predicted proteome of *Verrucomicrobium spinosum* and have only been found in the genomes of several Gram-negative bacteria that encode EpsH (exopolysaccharide locus protein H), including members of the PVC cluster (Haft et al., 2012). An EpsH-like gene is also found in the genome of *A. muciniphila* and upon further screening for the presence of PEP-CTERM proteins encoded by the genome of *A. muciniphila*, we found 23 predicted proteins containing this domain (Supplementary Table S4). The synthesis of 9 of these was confirmed by mass spectrometry, as they were present in at least one of the analyzed bacterial fractions. However, these appeared to be differentially regulated as some were only found in *A. muciniphila* grown on either glucose or mucin.

### Abundant OM Proteins and OM Location of the PilQ Protein in *A. muciniphila*

Three OM proteins (encoded by the genes with locus tags Amuc_0336, Amuc_1310 and Amuc_1098) were the most highly abundant in *A. muciniphila* grown on mucin or glucose, altogether making up to 20–40% of the total OM protein (Supplementary Table S2). BLAST analysis predicted the 17-kD protein encoded by the gene with locus tag Amuc_1310 to belong to a protein superfamily that includes several *Rickettsia* genus-specific 17 kD-surface antigen proteins, suggesting its potential interaction with the environment. The 82-kD protein encoded by the gene with locus tag Amuc_0336 could be annotated as a TonB-dependent receptor and showed low but significant similarity (sequence identity ~25%) to TonB-dependent receptors from *Pseudomonas* spp, which are OM proteins involved in heme and iron transport (Takase et al., 2000).

By far the most prominent protein, which was found to be over twofold more abundant than any other OM protein, is the product of gene with locus tag Amuc_1098, further termed PilQ. The 907-residue protein PilQ was initially annotated as a types II and III secretion system protein (see Supplementary Table S2) and contains a highly conserved pilus secretin domain at position 650-876, while its N-terminal part has a conserved domain at position 71-239 of a beta-barrel assembly machinery (BAM) containing three tetratricopeptide repeats. Highly similar homologues (25–40% identity) of the *A. muciniphila* PilQ are predicted to be produced by other Verrucomicrobia, such as *V. spinosum*, *Haloferula* sp. and *Lentisphaera araneosa*, the homologue of the latter being annotated as the type IV fimbrial biogenesis protein PilQ. These results strongly suggests that Verrucomicrobia, and specifically *A. muciniphila*, may produce type IV pili that are secreted in a process that involves the OM located secretin PilQ (Berry and Pelicic, 2015).

**FIGURE 4:**
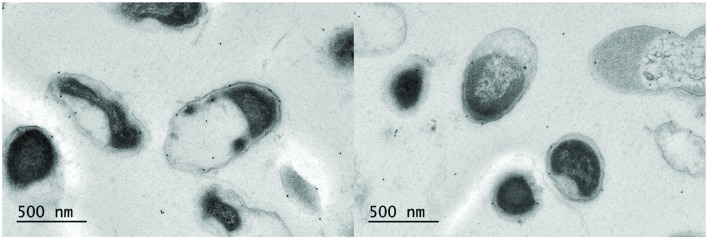
**Immunoelectron microscopy labeling of *A. muciniphila* thin sections visualizes PilQ to be localized on the OM.** The cells were labeled with anti-P-Amuc_1098A antibodies and detection was done by using 10-nm protein A-conjugated gold particles. Two representative electronmicrographs are shown.

Previous electron microscope (EM) images from *A. muciniphila* have revealed pili-like structures (Derrien et al., 2004). Therefore, we used immunogold labeling of *A. muciniphila* thin sections to determine the location of PilQ. The N-terminal domain of PilQ (termed P-Amuc_1098A) labeled with a C-terminal His-Tag was overproduced and purified from *E. coli* since the gene for the entire PilQ could not be stably maintained in the *E. coli* host Top10 (data not shown). Polyclonal rabbit antibodies against the purified termed P-Amuc_1098A were generated, labeled with protein A-conjugated gold particles, and used to treat thin sections of *A. muciniphila* cells that were subsequently examined by transmission electron microscopy (**Figure 4**). The results show that a major part of the protein A–gold particles were located in the OM, confirming the location of PilQ in the OM of *A. muciniphila.* Moreover, each thin section has various PilQ molecules, as is to be expected by the high level of abundance of the OM protein PilQ in *A. muciniphila.*

## Discussion

Using a combination of experimental mass-spectrometry based identification and advanced bioinformatics, we identified 79 putative OM proteins in *A. muciniphila*, which comprises 3.6% of the 2176 predicted protein-coding sequences in this mucus-degrading intestinal bacterium. This fraction is slightly higher than that found in other Gram-negative bacteria where it was estimated that 2–3% of the genome codes for OM proteins (Molloy et al., 2000; Wimley, 2003). As we included membrane-associated extracellular proteins in the analysis, the actual number of OM proteins in *A. muciniphila* is presumably somewhat lower. Extracellular proteins were included as they may be involved in communication with the host and are therefore of great interest. Seven complementary bioinformatic algorithms were applied to characterize *A. muciniphila* OM and extracellular proteins based on the predicted secretion signals, occurrence of transmembrane strands, presence of beta-barrel structures and attachment to the cell wall by lipid motifs. Moreover, the enrichment of the OM fractions was characterized by analysis of the activity of mucin-degrading enzymes in *A. muciniphila.* This included the sialidase activity, encoded by two secreted enzymes that recently were purified and characterized (Tailford et al., 2015).

Since it was discovered that sarkosyl can selectively solubilize cytoplasmic and inner membranes while conserving the integrity of the OM, this ionic detergent has been widely used in the purification of OM proteins in Gram-negative bacteria (Rhomberg et al., 2004; Brown et al., 2010; Moumene et al., 2015). In comparison to other methods, sarkosyl extraction leads to higher purity and better reproducibility of the OM extracts, while the basis for OM resistance to this detergent is not known (Filip et al., 1973; Frankel et al., 1991). In our study, sarkosyl-treatment was superior to the sucrose gradient centrifugation of *A. muciniphila* membranes and led to enrichment of OM proteins (**Figure 1**). Several proteins from other cellular locations were also present and the high sensitivity of LC-MS/MS might be one of the reasons why so many proteins were identified from the fractions. Hence, by optimizing the protocol, the abundance of non-OM proteins could be further reduced.

More insight into the OM proteome biosynthesis of *A. muciniphila* was generated through isolation and identification of proteins covering the most important pathways of OM biogenesis. The YaeT protein (the product of the gene with locus tag Amuc_1053), which is an essential protein for OM protein biogenesis due to its role in insertion of beta-barrel proteins into the OM (Werner and Misra, 2005; Tokuda, 2009), was among the 10 most highly abundant proteins in the extracted OM fractions. Furthermore, the organic solvent tolerance protein (encoded by the gene with the locus tag Amuc_1439), known also as increased membrane permeability (Imp) protein, needed for LPS transport to the bacterial cell surface (Bos et al., 2004), was found in high abundance. These results support the typical Gram-negative membrane architecture of *A. muciniphila*. However, we obtained no support for the presence of an intracytoplasmic membrane as is found in some PVC species (Lee et al., 2009). A recent study on the abundance of *A. muciniphila* in antibiotic-treated subjects has reported partial compartmentalization of some *A. muciniphila* cells (Dubourg et al., 2013). However, *A. muciniphila* does not contain the highly conserved genetic module, termed DUF1501, preferentially present in compartmentalized PVC species, nor the Planctomycetes and Verrumicrobia metabolosome genes (Kamneva et al., 2012).

The *A. muciniphila* protein PilQ was found to be the most highly produced OM protein in *A. muciniphila*, regardless of the growth conditions. The primary structure of the *A. muciniphila* PilQ includes a N-terminal BAM domain with three tetratricopeptide repeats, resembling the pilotin proteins that are essential for the folding and insertion of proteins in the OM of Gram-negative bacteria and involved in the biogenesis of type IV pili (Koo et al., 2013). Moreover, the *A. muciniphila* PilQ includes a C-terminal secretin domain, which is highly conserved in all secretins that allow passage through the OM of type IV pilins, long surface exposed filaments involved in a variety of functions, including motility and adherence to host cells (Berry and Pelicic, 2015). No atomic structures of secretins have been solved yet but advanced EM analysis has recently provided insight in the *Thermus thermophilus* and *Myxococcus xhantus* pilin machineries, showing that the PilQ of these bacteria form the OM channels as well as part of the periplasmic ring where type IV pili are being extruded (Burkhardt et al., 2011; Chang et al., 2016). The structure of the *A. muciniphila* PilQ is unique but conserved in proteins predicted to be produced by other Verrucomicrobia, including *V. spinosum*, *Haloferula* sp. and *L. araneosa*. We found the *A. muciniphila* PilQ to be enriched in the sarkosyl-resistant OM fraction, which is in line with the observation that the well-characterized *T. thermophilus* PilQ is resistant to sarkosyl treatment (Burkhardt et al., 2011). The immunogold labeling results presented here confirm the localization of PilQ in the OM of *A. muciniphila*. Based on this observation, the demonstrated OM location and the predicted structural homology of PilQ, we propose that the *A. muciniphila* PilQ is involved in the formation of type IV pili. The earlier observed protruding filaments in *A. muciniphila* could represent such type IV pili but further studies are needed to confirm this (Derrien et al., 2004). Hence, PilQ is an interesting candidate for immune signaling in *A. muciniphila*, as the type IV pili are known to be involved in a variety of interactions with the host (Ehara et al., 1993; Craig and Li, 2008; von Ossowski et al., 2013).

Other groups of OM proteins found to be produced by *A. muciniphila* were autotransporters, RND multidrug eﬄux pumps and YD proteins. Autotransporters are an extensive family of proteins, which can be either secreted or cell-surface-exposed, and function for example as enzymes, adhesins, cytotoxins or mediate bacterial motility (Grijpstra et al., 2013). They have mainly been studied for their virulence-related properties in pathogens but are also commonly found in non-pathogenic bacteria. RND multidrug eﬄux pumps exist in a tripartite form traversing both the OM and IM, and thus they are able to efficiently pump out drug molecules directly into extracellular space (Nikaido, 2011). The function of YD proteins is not very well studied but they have been suggested to be involved in carbohydrate binding and bacterial interactions with eukaryotic host cells (Jackson et al., 2009; Koskiniemi et al., 2013).

In addition to the OM proteins produced by *A. muciniphila*, we identified another 12 OM proteins from the genome using *in silico* analysis. These were not present in any of the studied fractions. Based on RNA-seq data all the genes encoding these proteins are transcriptionally active (Ottman, 2015), suggesting that their absence from the proteomics dataset could be due to a methodological reason, such as their low level production, high hydrophobicity, or post-translational modification. While we can presently not distinguish between these possibilities, we have been able to detect proteins that are post-translationally modified. These include the PEP-CTERM proteins that are involved in a specific sortase system and may be heavily post-translationally modified, including removal of N-terminal and C-terminal transmembrane domains and extensive glycosylation (Haft et al., 2012). *A. muciniphila* was predicted to encode 23 PEP-CTERM proteins and we detected nine of these by mass spectrometry. The proteins were present in relatively low abundance in the whole proteome samples, which may explain the difficulty in detecting them. Higher levels of PEP-CTERM proteins were detected in the intracellular fractions compared to OM fractions but we cannot exclude the possibility that this is related to different levels of glycosylation. While originally detected in *V. spinosum* but widely spread in the Gram-negatives, including the PVC cluster, peptides of the PEP-CTERM proteins have so far been experimentally identified in several Archaea (Haft et al., 2012). Hence, these findings suggest that *A. muciniphila* may be a well-suited model organism for studying the bacterial PEP-CTERM/exosortase system that so far has been little characterized.

Among the highly abundant proteins in the OM fractions were several proteins annotated as cytoplasmic, including six ribosomal proteins. It is possible that these proteins were so abundant in the cultures that they ended up in high concentrations also in the OM fraction, but it may also be that they have secondary roles in the bacteria. Proteins that are able to perform two or more functions are called moonlighting proteins (Copley, 2012). Several glycolytic, housekeeping and ribosomal proteins are often found on the surface of bacteria where they develop other functions (Henderson and Martin, 2011; Sherry et al., 2011).

The presence of specific OM proteins in Gram-negative bacteria is modulated by the available carbon and energy sources (Papasotiriou et al., 2008; Haussmann et al., 2009; Yang et al., 2011). The abundance of one third of the putative *A. muciniphila* OM proteins was altered during growth on glucose in comparison to mucin. However, for most of these proteins, the presence of other proteins with the same function was found in both conditions. This suggests that the specific function is not missing, but is taken over by a different protein. Unfortunately, many of the proteins that were most affected by the difference in carbon sources remain uncharacterized, despite efforts to search for related proteins. This is a common issue when handling proteomics data, especially as *A. muciniphila* belongs to the PVC superphylum, which has not been studied extensively yet. Further research into the function of these proteins could reveal important characteristics of *A. muciniphila*.

This study provides the first proteomic characterization of *A. muciniphila* OM proteins. We have identified highly abundant proteins involved in secretion and transport, as well as many uncharacterized proteins. In particular, we confirmed by immunoelectron microscopy the localization of the most abundant OM protein PilQ that is predicted to be involved in type IV pili formation by *A. muciniphila*.

## Author Contributions

NO, HS, CB, WdV conceived and designed the experiments. NO, LH, JR, SB, JK performed the experiments. NO, CB, WdV analyzed the data. NO and WdV drafted the manuscript and all authors contributed to the final article.

## Conflict of Interest Statement

The authors declare that the research was conducted in the absence of any commercial or financial relationships that could be construed as a potential conflict of interest.
