# Supplementary material for: Characterization of Outer Membrane Proteome of Akkermansia muciniphila Reveals Sets of Novel Proteins Exposed to the Human Intestine
Source: Front Microbiol. 2016 Jul 26;7:1157. doi: 10.3389/fmicb.2016.01157 (PMC4960237; doi:10.3389/fmicb.2016.01157)
Supplement: Supplementary file 1 [file Data_Sheet_1.DOCX]

Supplementary Material

**Characterization of Outer Membrane Proteome of *Akkermansia muciniphila* Reveals Sets of Novel Proteins Exposed to the Human Intestine**

**Noora Ottman^1,2^, Laura Huuskonen^3^, Justus Reunanen^3,4^, Sjef Boeren^5^, Judith Klievink^6^, Hauke Smidt^1^, Clara Belzer^1^ and Willem M. de Vos^1,3,6,*^**

^1^Laboratory of Microbiology, Wageningen University, Wageningen, the Netherlands

^2^Metapopulation Research Centre, University of Helsinki, Helsinki, Finland

^3^Department of Veterinary Biosciences, University of Helsinki, Helsinki, Finland

^4^Microbiology and Biotechnology, Department of Food and Environmental Sciences, University of Helsinki, Finland

^5^Laboratory of Biochemistry, Wageningen University, Wageningen, the Netherlands

^6^Department of Bacteriology and Immunology, and Research Programs Unit, Immunobiology, University of Helsinki, Helsinki, Finland

***Correspondence:**

Willem M. de Vos

willem.devos@wur.nl

# Supplementary Figures and Tables

## Supplementary Figures

**Supplementary Figure 1.** **Relative protein concentration of the sucrose-density gradient fractions for mucin and glucose-grown *A. muciniphila*.** 24 fractions were collected from each gradient and four fractions from each condition (black markers, marked M1-M4 and G1-G4) were selected for further analysis by mass spectrometry and enzyme activity.


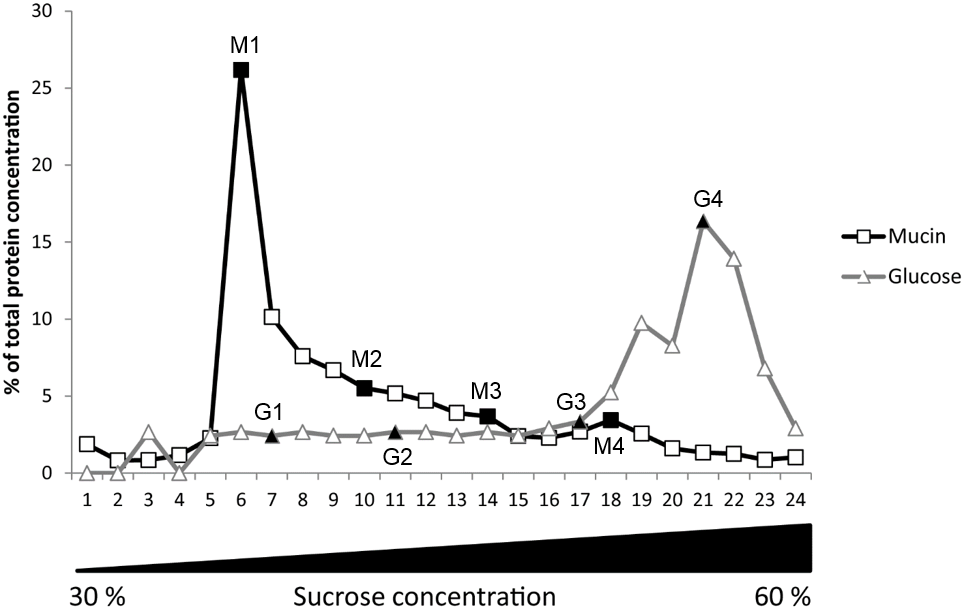


**Supplementary Figure 2. Prediction of outer membrane proteins.** All proteins identified from the sarkosyl-extracted OM fraction were subjected to the prediction approach for the identification of OM proteins. The total number of selected proteins for *A. muciniphila* grown on mucin (left) and glucose (right) are shown for each step.


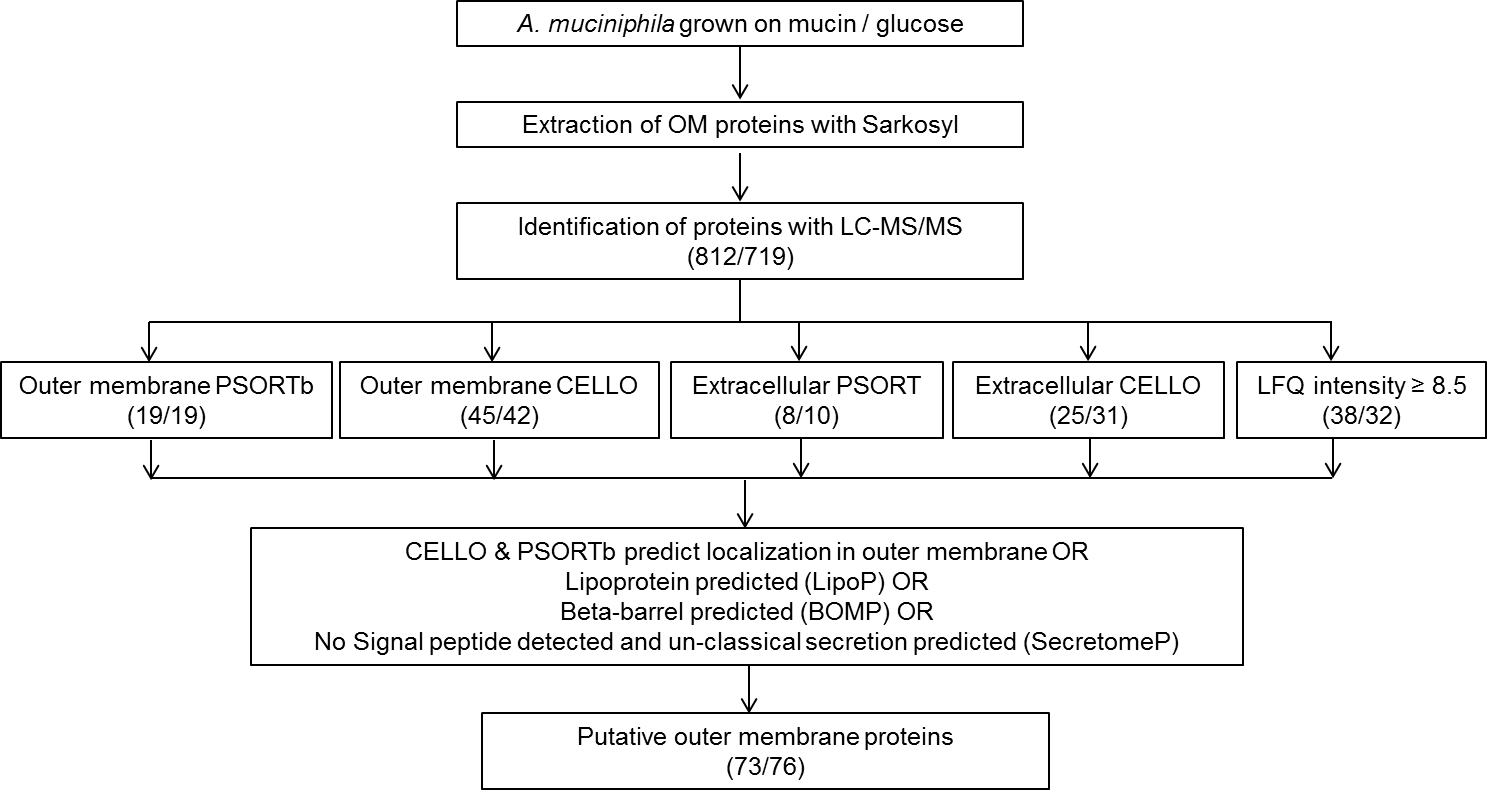


**Figure S3. Classification of OM proteins.** Proteins in the sarkosyl-extracted OM fraction of *A. muciniphila* grown on mucin (dark) or glucose (light) were identified by LC-MS/MS and classified based on analysis of their sequences. See Table S2 for a detailed comparison and description of all predicted functions.


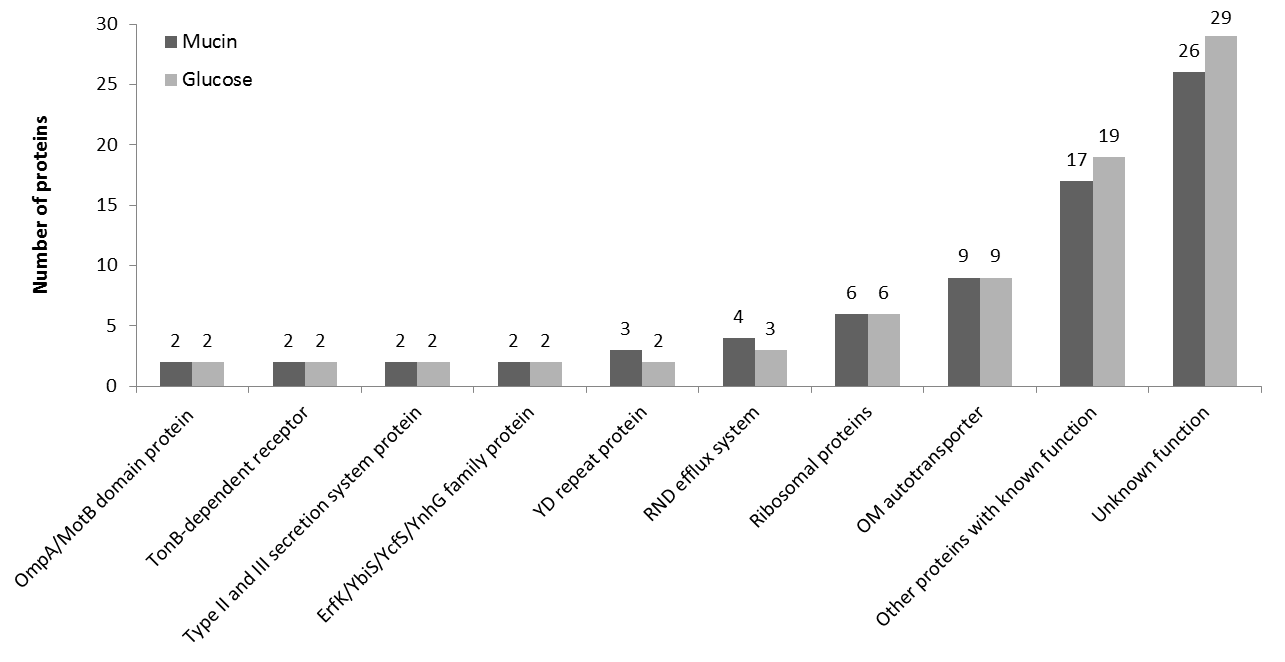


## Supplementary Tables

**Supplementary Table 1. Number of identified proteins from each bacterial fraction analysed by mass spectrometry.** Each fraction was extracted from either mucin or glucose grown *A. muciniphila* cultures. The amount of proteins detected from *A. muciniphila* whole proteome samples is shown for comparison.

|  | Number of proteins | |
| --- | --- | --- |
| Sample | Mucin | Glucose |
| Whole proteome | 1054 | 1087 |
| Sarkosyl method | 812 | 719 |
| Intracellular fraction | 965 | 999 |
| Sucrose density-gradient fraction 1 | 527 | 141 |
| Sucrose density-gradient fraction 2 | 517 | 325 |
| Sucrose density-gradient fraction 3 | 507 | 352 |
| Sucrose density-gradient fraction 4 | 488 | 439 |

**Supplementary Table 2. Putative OM and membrane-associated extracellular proteins.** A total of 79 proteins were identified as OM or extracellular proteins from sarkosyl-extracted OM fractions of *A. muciniphila* grown on mucin or glucose by using bioinformatics tools. Relative abundances of the proteins based on mass spectrometry analysis are presented on a log10 scale as LFQ intensities. Relative abundance of 4.0 represents proteins that were not detected or were under the detection limit. Proteins with a change of > 10-fold in relative abundance between extracts from bacteria grown on mucin and glucose are indicated in grey, and the most abundant OM proteins are indicated in boldface.

| Locus tag | Annotation | Glucose  (Log10 LFQ intensity) | Mucin  (Log10 LFQ intensity) | Fold change |
| --- | --- | --- | --- | --- |
| Amuc_0006 | Putative uncharacterized protein | 4.0 | 5.4 | 25.9 |
| Amuc_0019 | Putative uncharacterized protein | 5.4 | 7.4 | 109.6 |
| Amuc_0032 | Putative uncharacterized protein | 7.8 | 6.3 | 35.1 |
| Amuc_0036 | YD repeat protein | 5.9 | 6.8 | 7.2 |
| Amuc_0074 | Putative uncharacterized protein | 8.4 | 7.9 | 2.6 |
| Amuc_0105 | RND efflux system, outer membrane lipoprotein, NodT family | 7.9 | 8.7 | 5.8 |
| Amuc_0172 | Putative uncharacterized protein | 7.4 | 8.9 | 31.0 |
| Amuc_0194 | Putative uncharacterized protein | 8.6 | 8.2 | 2.7 |
| Amuc_0219 | Efflux transporter, RND family, MFP subunit | 6.4 | 6.2 | 1.7 |
| Amuc_0294 | 30S ribosomal protein S17 | 8.4 | 8.8 | 2.5 |
| Amuc_0301 | 50S ribosomal protein L2 | 8.3 | 8.7 | 2.7 |
| Amuc_0304 | 50S ribosomal protein L3 | 8.3 | 8.5 | 1.7 |
| Amuc_0308 | 30S ribosomal protein S12 | 8.0 | 8.7 | 5.4 |
| **Amuc_0336** | **TonB-dependent receptor** | **9.9** | **9.4** | **3.8** |
| Amuc_0355 | Putative uncharacterized protein | 8.5 | 8.1 | 2.8 |
| Amuc_0360 | Putative uncharacterized protein | 8.4 | 8.2 | 1.6 |
| Amuc_0371 | Two component regulator propeller domain protein | 5.7 | 7.4 | 48.5 |
| Amuc_0385 | ErfK/YbiS/YcfS/YnhG family protein | 6.6 | 8.1 | 31.0 |
| Amuc_0392 | Coagulation factor 5/8 type domain protein | 7.6 | 7.8 | 1.7 |
| Amuc_0394 | Putative uncharacterized protein | 7.6 | 7.8 | 1.7 |
| Amuc_0433 | Putative uncharacterized protein | 7.5 | 7.4 | 1.3 |
| Amuc_0435 | Putative uncharacterized protein | 8.4 | 8.8 | 2.4 |
| Amuc_0438 | Ribosomal protein S11 | 7.7 | 9.1 | 29.3 |
| Amuc_0513 | Putative uncharacterized protein | 8.5 | 7.6 | 7.3 |
| Amuc_0576 | Peptidase M16 domain protein | 7.5 | 8.8 | 19.7 |
| Amuc_0584 | Outer membrane autotransporter barrel domain protein | 8.7 | 7.5 | 16.4 |
| Amuc_0609 | Putative uncharacterized protein | 8.4 | 7.9 | 3.0 |
| Amuc_0610 | Tetratricopeptide TPR_2 repeat protein | 8.6 | 8.5 | 1.4 |
| Amuc_0682 | OmpA/MotB domain protein | 7.1 | 6.9 | 1.4 |
| Amuc_0687 | Outer membrane autotransporter barrel domain protein | 9.6 | 8.5 | 15.2 |
| Amuc_0735 | YD repeat protein | 4.0 | 5.8 | 67.4 |
| Amuc_0789 | Putative uncharacterized protein | 7.2 | 4.0 | 1669.9 |
| Amuc_0815 | DNA polymerase III, beta subunit | 6.5 | 6.2 | 1.9 |
| Amuc_0820 | Peptidoglycan-binding LysM | 8.9 | 8.9 | 1.0 |
| Amuc_0823 | Putative uncharacterized protein | 7.8 | 7.8 | 1.1 |
| Amuc_0837 | Putative uncharacterized protein | 6.1 | 4.0 | 125.7 |
| Amuc_0904 | Aconitate hydratase 1 | 6.2 | 5.5 | 4.5 |
| Amuc_0931 | 50S ribosomal protein L15 | 8.4 | 9.0 | 3.3 |
| Amuc_0967 | RNP-1 like RNA-binding protein | 7.5 | 7.7 | 1.7 |
| Amuc_1008 | Glycoside hydrolase family 31 | 6.2 | 6.3 | 1.5 |
| Amuc_1026 | Peptidyl-prolyl cis-trans isomerase | 6.7 | 4.0 | 542.7 |
| Amuc_1039 | Outer membrane autotransporter barrel domain protein | 8.1 | 6.0 | 134.5 |
| Amuc_1053 | Outer membrane protein assembly complex, YaeT protein | 9.4 | 9.2 | 1.5 |
| Amuc_1061 | Outer membrane protein-like protein | 8.0 | 8.4 | 2.1 |
| **Amuc_1098** | **Type II and III secretion system protein** | **10.3** | **9.8** | **3.0** |
| Amuc_1114 | Outer membrane autotransporter barrel domain protein | 6.5 | 7.0 | 3.4 |
| Amuc_1283 | Outer membrane autotransporter barrel domain protein | 9.2 | 8.7 | 2.8 |
| **Amuc_1310** | **17 kDa surface antigen** | **10.0** | **9.6** | **2.2** |
| Amuc_1333 | Putative uncharacterized protein | 7.0 | 4.0 | 931.8 |
| Amuc_1382 | Secretion protein HlyD family protein | 7.0 | 6.4 | 3.6 |
| Amuc_1412 | Putative uncharacterized protein | 9.5 | 9.2 | 1.9 |
| Amuc_1420 | Putative uncharacterized protein | 5.5 | 5.7 | 1.6 |
| Amuc_1434 | Putative uncharacterized protein | 7.4 | 7.5 | 1.1 |
| Amuc_1439 | Organic solvent tolerance protein OstA-like protein | 8.6 | 8.3 | 2.1 |
| Amuc_1500 | Polysaccharide deacetylase | 9.0 | 9.2 | 1.9 |
| Amuc_1512 | OmpA/MotB domain protein | 6.1 | 5.8 | 2.2 |
| Amuc_1514 | Putative uncharacterized protein | 6.7 | 6.4 | 1.8 |
| Amuc_1525 | Putative uncharacterized protein | 6.6 | 6.7 | 1.2 |
| Amuc_1537 | Outer membrane autotransporter barrel domain protein | 8.7 | 8.2 | 3.3 |
| Amuc_1583 | Putative uncharacterized protein | 7.0 | 7.0 | 1.1 |
| Amuc_1620 | Outer membrane autotransporter barrel domain protein | 7.6 | 6.3 | 20.7 |
| Amuc_1638 | Type II and III secretion system protein | 9.4 | 8.9 | 3.7 |
| Amuc_1656 | Putative uncharacterized protein | 9.8 | 9.5 | 2.0 |
| Amuc_1684 | TonB-dependent receptor | 8.9 | 8.0 | 8.4 |
| Amuc_1687 | Putative uncharacterized protein | 9.4 | 9.0 | 2.4 |
| Amuc_1722 | Outer membrane autotransporter barrel domain protein | 9.2 | 8.0 | 15.0 |
| Amuc_1723 | Outer membrane autotransporter barrel domain protein | 8.3 | 6.6 | 44.6 |
| Amuc_1743 | Putative uncharacterized protein | 8.8 | 8.8 | 1.2 |
| Amuc_1891 | Putative uncharacterized protein | 9.6 | 6.6 | 909.0 |
| Amuc_1939 | Efflux transporter, RND family, MFP subunit | 4.0 | 5.9 | 79.6 |
| Amuc_2043 | RND efflux system, outer membrane lipoprotein, NodT family | 9.2 | 9.2 | 1.1 |
| Amuc_2077 | Polysaccharide export protein | 9.0 | 8.3 | 5.4 |
| Amuc_2099 | Putative uncharacterized protein | 8.5 | 8.5 | 1.1 |
| Amuc_2107 | Putative uncharacterized protein | 8.2 | 8.8 | 3.6 |
| Amuc_2108 | Glycoside hydrolase family 16 | 5.9 | 4.0 | 87.2 |
| Amuc_2111 | ErfK/YbiS/YcfS/YnhG family protein | 7.8 | 7.8 | 1.0 |
| Amuc_2127 | Carbohydrate-selective porin OprB | 9.1 | 8.5 | 4.4 |
| Amuc_2152 | YD repeat protein | 6.3 | 6.8 | 3.2 |
| Amuc_2165 | Putative uncharacterized protein | 5.9 | 4.0 | 76.7 |

**Supplementary Table 3. Putative OM proteins predicted and detected.** A total of 17 proteins were predicted to be OM proteins but were not detected by mass spectrometry in the OM fractions. However, the proteins Amuc_0356 and Amuc_0480 were detected in the sucrose density-gradient samples and Amuc_0983, Amuc_1011 and Amuc_1945 were detected in the whole proteome samples.

| Locus tag | Annotation |
| --- | --- |
| Amuc_0146 | Alpha-L-fucosidase |
| Amuc_0173 | Uncharacterized protein |
| Amuc_0356 | Uncharacterized protein |
| Amuc_0434 | Uncharacterized protein |
| Amuc_0480 | Alpha-1,3-galactosidase B |
| Amuc_0599 | Uncharacterized protein |
| Amuc_0892 | Outer membrane autotransporter barrel domain protein |
| Amuc_0908 | Uncharacterized protein |
| Amuc_0983 | YD repeat protein |
| Amuc_1011 | Uncharacterized protein |
| Amuc_1123 | Uncharacterized protein |
| Amuc_1143 | YD repeat protein |
| Amuc_1145 | Uncharacterized protein |
| Amuc_1340 | Uncharacterized protein |
| Amuc_1350 | Uncharacterized protein |
| Amuc_1831 | Uncharacterized protein |
| Amuc_1945 | Uncharacterized protein |

**Supplementary Table 4. PEP-CTERM domain-containing proteins predicted and detected.** A total of 23 proteins were found to contain a PEP-CTERM domain in the genome of *A. muciniphila*. All the proteins are uncharacterized. Proteomic evidence was detected for nine of these. Relative abundance of the protein on a log10 scale in the fractions is shown. ND; not detected.

| **Locus tag** | **Mucin** | | | **Glucose** | | |
| --- | --- | --- | --- | --- | --- | --- |
|  | **Whole proteome** | **Intracellular** | **Outer membrane** | **Whole proteome** | **Intracellular** | **Outer membrane** |
| Amuc_0215 | ND | ND | ND | ND | ND | ND |
| Amuc_0428 | ND | ND | ND | ND | ND | ND |
| Amuc_0622 | ND | ND | ND | ND | ND | ND |
| Amuc_0646 | 6.49 | 6.62 | ND | 6.49 | 7.32 | 6.21 |
| Amuc_0688 | ND | ND | ND | ND | ND | ND |
| Amuc_0789 | 6.93 | 7.82 | ND | 5.84 | 8.35 | 7.22 |
| Amuc_0825 | ND | ND | ND | ND | ND | ND |
| Amuc_0952 | ND | 6.97 | ND | 5.05 | 7.59 | 5.70 |
| Amuc_0990 | 4.63 | 5.35 | ND | 5.57 | 6.78 | ND |
| Amuc_0991 | ND | ND | ND | ND | ND | ND |
| Amuc_1028 | ND | ND | ND | ND | ND | ND |
| Amuc_1209 | ND | ND | ND | ND | ND | ND |
| Amuc_1221 | ND | ND | ND | ND | ND | ND |
| Amuc_1266 | ND | ND | ND | ND | ND | ND |
| Amuc_1333 | 6.81 | 7.39 | ND | 6.61 | 7.94 | 6.97 |
| Amuc_1451 | ND | ND | ND | 4.54 | 7.02 | ND |
| Amuc_1560 | ND | ND | ND | ND | ND | ND |
| Amuc_1978 | ND | ND | ND | ND | ND | ND |
| Amuc_1982 | ND | ND | ND | ND | ND | ND |
| Amuc_2045 | ND | 5.48 | ND | 6.16 | 6.68 | ND |
| Amuc_2105 | ND | ND | ND | ND | ND | ND |
| Amuc_2126 | 4.66 | 5.83 | ND | 5.51 | 6.84 | ND |
| Amuc_2165 | ND | 7.35 | ND | 4.77 | 7.79 | 5.88 |

**
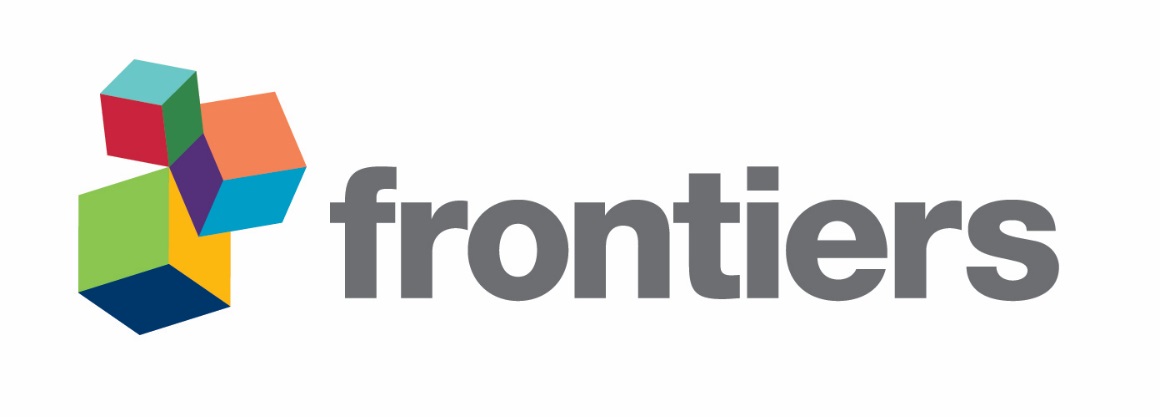
**
